# Phosphorus Recycling from an Unexplored Source by Polyphosphate Accumulating Microalgae and Cyanobacteria—A Step to Phosphorus Security in Agriculture

**DOI:** 10.3389/fmicb.2015.01421

**Published:** 2015-12-22

**Authors:** Chandan Mukherjee, Rajojit Chowdhury, Krishna Ray

**Affiliations:** Environmental Biotechnology Group, Department of Botany, West Bengal State UniversityKolkata, India

**Keywords:** global food security, parboiled rice mill effluent, phosphorus pollution, microalgae and cyanobacteria, polyphosphates, phosphorus biofertilizers, phosphorus scarcity

## Abstract

Phosphorus (P), an essential element required for crop growth has no substitute. The global food security depends on phosphorus availability in soil for crop production. World phosphorus reserves are fast depleting and with an annual increase of 2.3% in phosphorus demand, the current reserves will be exhausted in coming 50–100 years. India and other Western countries are forced to import phosphorus fertilizers at high costs to meet their agricultural demands due to uneven distribution of phosphate rocks on earth. The present study from India, aims to draw attention to an unnoticed source of phosphorus being wasted as parboiled rice mill effluent and subsequent bio-recovery of the valuable element from this unconventional source. The research was conducted in West Bengal, India, a state with the highest number of parboiled rice mills where its effluent carries on an average ~40 mg/L of soluble phosphorus. Technology to recover and recycle this wastewater P in India in a simple, inexpensive mode is yet to be optimized. Our strategy to use microalgae, *Chlorella* sp. and cyanobacteria, *Cyanobacterium* sp., *Lyngbya* sp., and *Anabaena* sp. to sequester the excess phosphorus from the effluent as polyphosphate inclusions and its subsequent recycling as slow and moderate release phosphorus biofertilizers to aid plant growth, preventing phosphorus loss and pollution, is a contemporary venture to meet the need of the hour. These polyphosphate accumulating microorganisms play a dual role of remediation and recovery of phosphorus, preliminarily validated in laboratory scale.

## Global phosphorus scenario

Phosphorus (P), the 11th most abundant element found in earth's crust, is necessary for survival of life, as it is the main backbone of DNA, RNA, and ATP, the key components of a living cell (Cordell and White, 2011). Phosphorus is a limiting nutrient for crop growth having no substitute and hence, food security worldwide depends on the P availability in soil for crop production (Cordell et al., 2011). In most soils, the amount of available P is ~1 μmol/L but requirement estimated is ~30 μmol/L (Adhya et al., 2015). Eighty two percent of the phosphate rocks, a non-renewable source of P which takes around 10–15 million years to form (Cordell et al., 2009) are mined globally and added to soil as P fertilizers to address the problem of P scarcity in soil for optimum crop production (Adhya et al., 2015). Around 0.007% of the total P present on the earth's crust (4 × 10^6^ billion metric tons) is found as phosphate rock resources which contain about 5–13% P, out of which only 20% reserves can be exploited by mining economically (Cordell and White, 2011). Present estimations predict the total phosphate rock resources to be around 300 billion metric tons (MT) and the reserves as 71 billion MT (Subba Rao et al., 2015). These P reserves are fast dwindling and with an increase of 2.3% per annum in annual P demands (Adhya et al., 2015), the current reserves are expected to be depleted in the coming 50–100 years (Cordell et al., 2009).

India and other Western European countries are totally dependent on imports to meet their P demands (Cordell et al., 2009) because the distribution of phosphate rocks is uneven with Morocco having approximately 85% of the global share, followed by China with 6% and the US with 3% (Obersteiner et al., 2013). India imports around 90% of its P fertilizer requirement to overcome P deficiency in soil and enhance crop production to meet the demands of its fast growing population (Bagyaraj et al., 2015) and is the largest importer of phosphate rocks in the world, importing about 30% of the total world trade (Subba Rao et al., 2015). The consumption of P fertilizer in India increased from 5.3 × 10^4^ MT in 1960–1961 to 7.3 × 10^6^ MT in 2009–2010 and is expected to reach 1.4 × 10^7^ MT by 2030–2031 (Abrol et al., 2015). An estimated 8.1 million MT of P_2_O_5_ (i.e., phosphorus fertilizer) was consumed in Indian agriculture in 2010–2011 to produce 235 million MT of food (Subba Rao et al., 2015). It is predicted that the food grain necessity of India will cross 300 million MT by the year 2025, compelling the use of around 13.1 million MT of P_2_O_5_ as fertilizer for crop production and leading to huge monetary loss of INR 7.81 billion (Elanchezhian et al., 2015).

On the other hand, approximately 1.9 × 10^7^ MT of P is mined per annum from phosphate rocks for application to soil but only one- fifth of this amount really reaches the consumers (Cordell et al., 2011) and the rest is lost at various stages, with up to 80% of loss occurring due to erosion of agricultural soils (Obersteiner et al., 2013), culminating in eutrophication of water bodies. Processes need to be planned to minimize losses and recycle P from agricultural lands (estimated at around 8 MT P) and the food commodity chain (estimated at around 2 MT P) (Cordell et al., 2009). An integrated approach is needed to overcome the problems of phosphorus dearth and over-use of phosphorus fertilizers causing pollution of soil and water.

Recovery of inorganic phosphorus into phosphates of calcium, iron, aluminum and magnesium-ammonium (struvite) from wastewater or sludge is yet to be widely accepted in developing countries, while the direct use of wastewater or waste sludge for agriculture is not recommended because of its associated toxic compounds like heavy metals, etc. (Sartorius et al., 2012). New techniques should be designed to extract P from alternative renewable phosphorus sources, like manure (around 15 MT P), human excreta (around 3 MT P) and food residues (around 1.2 MT P) (Cordell et al., 2009). New phosphorus sources should be investigated to recycle P, e.g., the animal bone wastes in Ethiopia could generate 28–58% of the annual phosphorus fertilizer supplies over the period 2008–2011 (Simons et al., 2014). Our research presented here falling in the same line, aims to draw attention to an unheeded point source of P pollution in the environment—the parboiled rice mill effluent (RME), from where phosphorus can be recovered and used in future as biofertilizers for plant growth improvement.

## The context of parboiled rice mill effluent as a phosphorus source

In the parboiling process, paddy is soaked in water and subsequently steamed and dried, before milling. This helps to minimize the breakage of rice and reduce the loss of nutrients during milling (Rathnayake et al., 2010). The effluent generated after parboiling contains a high load of pollutants including phosphorus and is discharged in the nearby land and water bodies increasing pollution of soil and surface water. The source of this high amount of P in RME is phytic acid (inositol hexakisphosphate, IP6), or phytate in its salt form. It is a saturated cyclic acid and principal storage form of phosphorus in rice brans. Phytase is the enzyme that catalyzes hydrolysis of phytic acid and releases a usable form of inorganic phosphorus. Soaking of paddy before processing by parboiled rice millers stimulates phytase of grains to hydrolyze stored phytate (Faria et al., 2006), releasing a great quantity of inorganic phosphorus in water, and subsequently increasing the inorganic phosphorus load of parboiled RME.

Official statistical data about P content of parboiled RME is surprisingly lacking in published literature and therefore escaped global attention, despite its “highly polluted status” earned because of high COD (437–4500 mg/L), BOD (211–2223 mg/L) and TSS (62–1258 mg/L) as reported by public funded research in India (CPCB, 2007; Asati, 2013; Haridas, 2013). The P content of rice mill effluent from U.S. and Brazil was reported to be 98 mg/L (EPA, 1974) and 34–143 mg/L (Faria et al., 2006), respectively. In United States, rice mill industry uses 1400–2100 liters of water per MT of rice in addition to water used in boilers (EPA, 1974). In Sri Lanka, up to 604 × 10^3^ L of RME is discharged to the environment per 8 MT of soaked paddy without being treated (Rathnayake et al., 2010). From Brazil, the volume of RME is estimated to be 2000 L per MT of rice, equivalent to 5.04 × 10^11^ L of effluent per year (de los Santos et al., 2012).

Based on our study conducted on a representative state of India, West Bengal, with highest production of rice (12.43 × 10^6^ MT per year) where more than 16925 parboiled rice mills are operational (CPCB, 2008), we observed that phosphorus content lies in the range of 30–72 mg/L in the effluent. The average concentration of soluble phosphorus in the RME samples collected from West Bengal is ~40 mg/L. In India, the lowest possible average outflow of effluent from each parboiling rice mill is about 100,000 L/day (Varshney, 2012). Assuming this, total soluble phosphorus wasted will amount to 4 kg/day by a single rice mill. Thus, in West Bengal alone approximately 67,700 kg (i.e., 67.7 MT) soluble phosphorus is wasted per day. If we presume that there are 340 working days in a parboiled rice mill per annum, then only in the state of West Bengal, India about 23,018 MT of soluble phosphorus is wasted per year. The latest price of superphosphate fertilizer is INR 25,244 per MT (Indexmundi, 2015). Superphosphate contains 45% P_2_O_5_ or 19.8% soluble phosphorus in it. So, the price of 198 kg soluble phosphorus is INR 25,244. Therefore, almost INR 3 billion is wasted in the form of soluble phosphorus present in RME discharged per annum in West Bengal only. The total volume of RME generated in India amounts to 20 × 10^6^ L per annum per rice mill (Varshney, 2012) and so, for approximately 57,850 parboiled rice mills in India (CPCB, 2008), the actual figure per annum becomes too large, resulting in a massive loss for Indian economy in the current perspective and an enormous waste burden on Indian environment.

There are definite guidelines existing in India for the level of pollutants in RME discharge but no minimum permissible limit for P is assigned. According to the Environment (Protection) Act of India, 1986 of Central Pollution Control Board (CPCB), every rice mill is supposed to have a functional full-fledged Effluent Treatment Plant (ETP) consisting of biological treatment process (CPCB, 2007). Due to the high cost of establishment and maintenance of the existent ETP, a majority of rice mills in India flout the CPCB guidelines (Business Standard, 2015; Paul et al., 2015). Under this paradox, our group established a simplified low-cost phosphorus recovery process by assimilation of this excess phosphorus into insoluble polyphosphate through luxury uptake of scavenger microalgae and cyanobacteria (Ray et al., 2013). The single aerobic free flow tank unit for treatment of RME, biomass harvest after P recovery, followed by release of the treated effluent to adjacent agricultural plots is a simpler and cheaper technique than the elaborate ETP consisting of four operational units, even considering the annual operational and maintenance cost after its installation.

Upflow Anaerobic Sludge Blanket (UASB) reactor (Haridas, 2013) and Enhanced Biological Phosphorus Removal (EBPR), two key recognized technologies to bio-remediate wastewater are not considered appropriate for P-removal and recovery from RME. UASB accounts for negligible removal of phosphorus and results in consequent increase of P concentration under anaerobic conditions (de los Santos et al., 2012; Khan et al., 2013). The stringent criteria and complexity of EBPR with its high establishment cost rendered it unsuitable for parboiled rice mill industry in India. Moreover, the wastewater sludge generated in EBPR is bulky and mostly contaminated with heavy metals, harmful pathogens and other toxic substances which interfere with crop growth and often not recommended to be used as agricultural fertilizer (Sartorius et al., 2012; Yuan et al., 2012). On the contrary, the proposed strategy simply resembles the growth of microalgae and cyanobacteria in eutrophicated water bodies. Our test microorganisms grow in RME in an open free flow tank under tropical environment and once introduced, out-compete other organisms native to RME and over-populate the effluent within a short time, while their biomass can be used as biofertilizers without any risk of contamination from external sources.

## Microalagae and cyanobacteria—phosphorus sequestration as polyphosphates

Polyphosphates (poly-P) are linear polymers containing tens to hundreds of orthophosphate residues linked by phosphoanhydride bonds. Poly-P is a very rich source of energy supporting growth and survival of organisms in adverse situations for a long period. Cyanobacteria and microalgae grow and uptake inorganic phosphorus and store it within their cells as poly-P granules to cope with unfavorable conditions like salt stress, osmotic stress, UV radiation, and fluctuations of pH and temperature in the environment (Achbergerová and Nahalka, 2011). Since last few years, microalgal species like *Chlorella* sp. and *Scenedesmus* sp. and cyanobacterial species like *Aphanothece* sp., *Spirulina* sp., *Arthrospira* sp., and *Phormidium* sp. have been used extensively in removal of nutrients from wastewater (Ray et al., 2013). The novelty of present research lies in the concept that microalgae and cyanobacteria are not only used for excess P removal from RME, but also the assimilated phosphorus as poly-P in their cells is utilized in soil as slow and moderate release phosphorus biofertilizers to optimize plant growth. The release of plant available phosphorus from the insoluble poly-P present in the biomass depends on the activity of phosphorus-solubilizing organisms (PSOs) existing in the soil, making the whole process very slow and steady and thus, supplying P within the “critical value” for crops in the rhizosphere. It reduces the probability of excess P supply (Ray et al., 2013) and controls consequent escape of P as soil run-off originating from unrestrained use of inorganic fertilizers.

## Phosphorus remediation and recycling from parboiled RME—A laboratory scale endeavor

**Figure 1:**
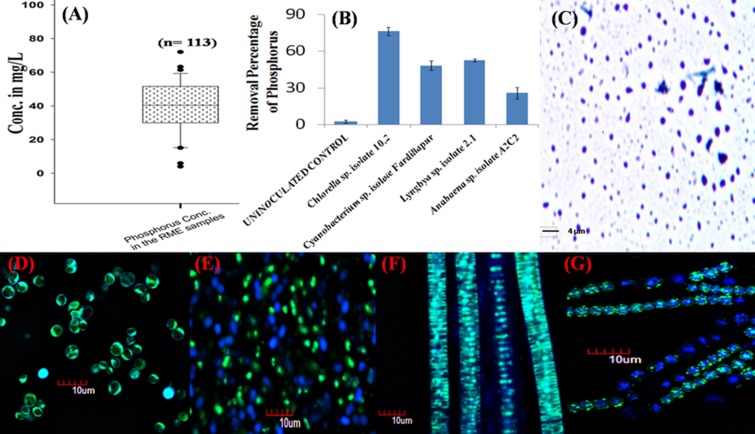
**Soluble phosphorus in parboiled rice mill effluent and its remediation by microalgae and cyanobacteria as polyphosphate accumulators. (A)** Box plot showing the phosphorus concentration in the rice mill effluent samples collected from West Bengal, India. “n” is the number of rice mills from where the samples were collected. Solid line in the box represents median values. Box represents 25–75% percentiles; range bar represents 5 and 95% percentiles, and dots beyond these bars represent values outside the 95% confidence interval. **(B)** Percentage removal of phosphorus from a rice mill effluent sample (initial phosphorus concentration was 35 mg/L) by different microalgae and cyanobacteria over a period of 21 days. All the bars on the graph represent the average data of 10 replicate experiments. Error bars were calculated on the basis of standard deviation of the data using the software Microsoft Excel. **(C)** Cell-free extract of polyphosphate granules stained with the Toluidine Blue dye as observed under bright field microscope. **(D–G)** DAPI staining of polyphosphate granules present in *Chlorella* sp. isolate 10.2 (Accession No. KJ654316), *Cyanobacterium* sp. isolate Fardillapur (Accession No. JX023443), *Lyngbya* sp. isolate 2.1 (Accession No. KF644563) and *Anabaena* sp. isolate A2C2 (Accession No. KF644564) observed under confocal microscope. The yellowish-green fluorescence indicates the presence of polyphosphate granules in the cells whereas the cells devoid of the granules emit blue fluorescence.

In the present study, RME samples were collected from 113 different parboiled rice mills located in three districts of West Bengal, India (33 from Hooghly, 50 from Burdwan, and 30 from Birbhum). The initial inorganic P level in the effluent was measured by Molybdenum Blue method (Krishnaswamy et al., 2009) (Figure 1A). Four environmental isolates comprising of three cyanobacterial genera, *Cyanobacterium* sp. isolate Fardillapur (Accession No. JX023443), *Lyngbya* sp. isolate 2.1 (Accession No. KF644563) and *Anabaena* sp. isolate A2C2 (Accession No. KF644564), and one microalgal genus, *Chlorella* sp. isolate 10.2 (Accession No. KJ654316) established earlier from different P-rich eutrophicated niches (Ray et al., 2013), were tested for their growing capability in highly acidic RME (pH 4.2). 0.02 g of each pure culture isolate was inoculated in 15 ml of the different RME samples collected and incubated for 24 days at 28°C under 12:12 h light: dark conditions. After 24 days, the accumulated poly-P was extracted and quantified from equal dry weight of all the cyanobacterial and microalgal cells following an established protocol standardized in our laboratory (Mukherjee and Ray, 2015a). The cell free extract of poly-P granules were stained by Toluidine Blue dye using Albert's staining method (Albert, 1920) (Figure 1C). The poly-P granules within the cells were visualized by confocal microscopy after staining with DAPI (Figures 1D–G) following a standardized protocol developed in our laboratory (Mukherjee and Ray, 2015b).

In a laboratory scale study, 5 liter of RME in an aerobic free flow system with light source was inoculated separately with ~5 g biomass of 25 days old culture of the microalgae and cyanobacteria along with one un-inoculated control. The soluble P level and growth trend was recorded at an interval of every 3 days till 21 days and the final removal percentage of P from RME at the end of 21 days, by microalgae and cyanobacteria was estimated (Figure 1B). After 21 days, microalgal and cyanobacterial biomass was harvested and dried. The average weight of the harvested microalgal and cyanobacterial biomass increased from ~5 to 50 g after bioremediation of RME. Approximately 84 mg of poly-P was accumulated on an average by the microalgal and cyanobacterial strains from the initial 175 mg of soluble P present in the 5 liter RME. Thus, the net recovery percentage of P from the RME in the form of poly-P through luxurious P-assimilation by experimental organisms is 48% and soluble phosphorus worth almost INR 1.44 billion is expected to be recovered per annum in West Bengal only.

Two gram of dried biomass of each cyanobacteria and microalgae (containing poly-P) was mixed with 5 kg of non-sterile soil (pH-7.13) in separate pots. The soil had 0.3% organic carbon and contained 17.13 mg of soluble P, 12 mg nitrate-nitrogen and 7 mg ammonium-nitrogen, per kg of soil. Similarly, 575 mg of superphosphate and NPK (20: 20: 13) was mixed with 5 kg of soil following the standard recommended dose. The pots were sown with rice seedlings and watered at regular intervals and incubated at a constant 30°C temperature and 60% humidity in a greenhouse for 115 days under 12:12 h light: dark conditions.

**Figure 2:**
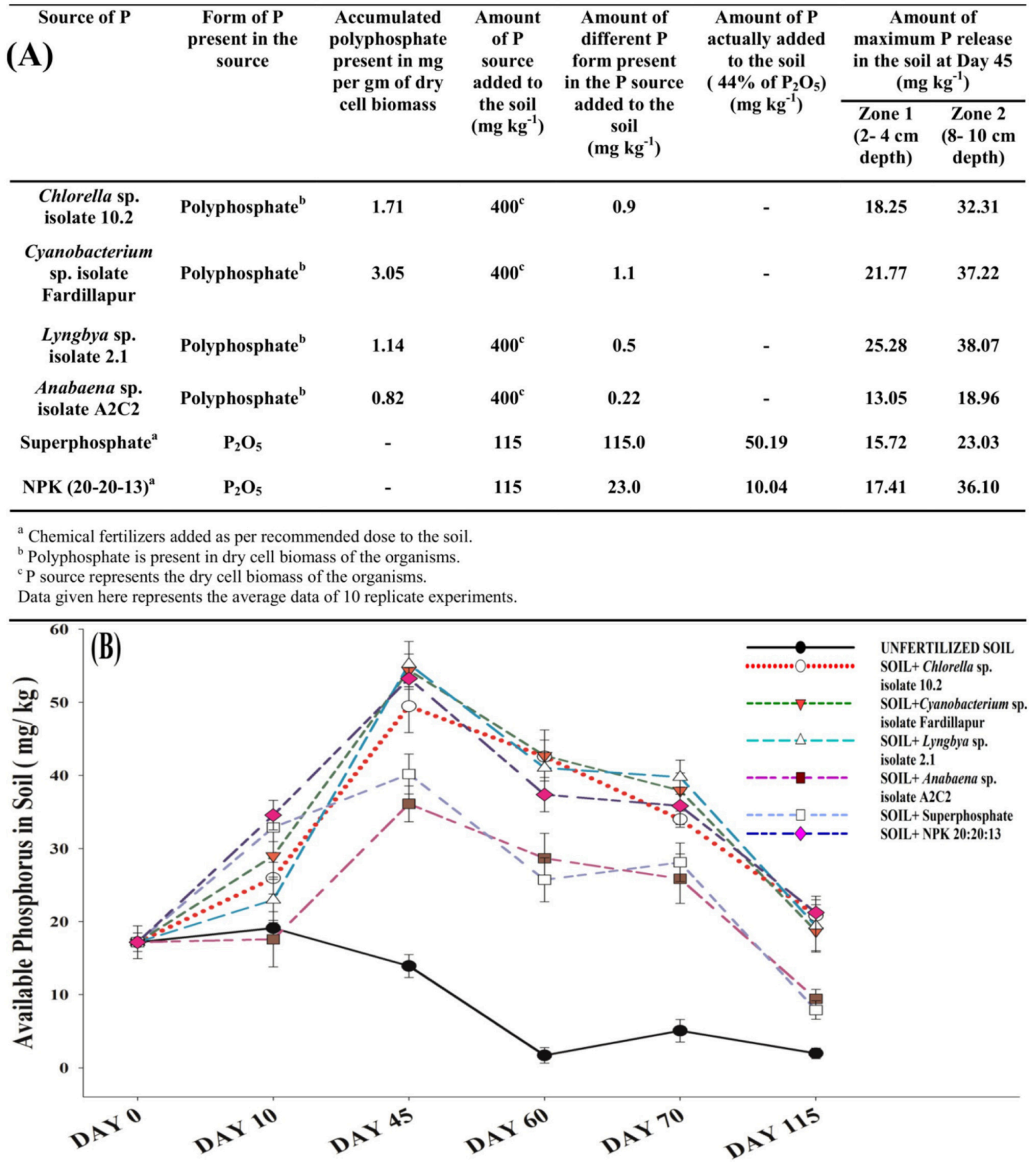
**Recycled polyphosphates as substitute to phosphorus fertilizers. (A)** Table showing the rate of conversion of polyphosphates accumulated by the microalgae and cyanobacteria into soluble phosphorus and comparison of its release to conventional chemical phosphorus fertilizers commercialized widely in India. **(B)** Polyphosphate releases soluble phosphorus at a comparable (maximum at 45 days) but slower rate (as reflected in initial 10 days) with recommended dose of superphosphate and NPK at 8–10 cm depth of soil. All the points on the graph represent the average data of 10 replicate experiments. Error bars were calculated on the basis of standard deviation of the data using the software SigmaPlot 13.0.

The net increase in plant available P concentration in soil recorded at different time intervals signified the release of soluble phosphorus from the insoluble poly-P, at 2–4 cm and 8–10 cm depth of soil as depicted in Figure 2A. The release of P from the poly-P was significantly comparable with the release of phosphorus from the commercial phosphate fertilizers- superphosphate and NPK (20: 20: 13). The biomass added to the non-sterile soil gets degraded by soil decomposers and its poly-P reservoir is exposed. Organic acids and phosphatases released by PSOs present in the rhizospheric zone liberate inorganic plant available P from polyphosphates (Gopalakrishnan et al., 2012; Sharma et al., 2013), leading to a slow but steady increase of soluble phosphorus content in soil over 45 days vis-à-vis the non-sterile soil supplemented with recommended dose of conventional P-fertilizers and also without any fertilizer as comparative control (Figure 2B).

A slower release of P from poly-P rich biomass is evident from the initial 10 days' slope (Figure 2B) in contrast to the commercial chemical P fertilizers which releases P readily after their application to the soil. The fall in soil test phosphorus (STP) after 45 days in soil supplemented with biomass was also observed to be more gradual (Figure 2B) than conventional fertilizers, indicating more persistent release of P. In addition, the released soluble P should be available in the rhizospheric region of the crops. The maximum rhizosphere zone length of rice is reported to be 10–15 cm (Gopalakrishnan et al., 2012). In all 10 replicate experiments for P release, maximum release was observed at 8–10 cm depth of soil (Figure 2A) and the result is presented graphically in Figure 2B. Thus, the mode of P release from poly-P from the decaying microalgal and cyanobacterial biomass is entirely a soil microbe-dependent natural phenomenon.

## The future outlook

In conclusion, the present venture provides a new avenue in the research area of polyphosphate granule accumulation by cyanobacteria and microalgae wherein these organisms play a dual role of bioremediation and recovery of phosphorus which was being wasted and its subsequent application as biofertilizers for crop growth. The present work can pave way for curbing the P shortage problem in low and middle income countries like India, Sri Lanka, Brazil, etc. where rice is the staple food crop and cut down imports of P fertilizers. Further studies are required to enhance the ability of the studied organisms to sequester more P from the RME and more P hyper-accumulating species have to be identified having high bioremediation capabilities. A large scale application of this viewpoint in parboiled rice mill crowded areas could be a much sought after goal to make this kind of P-recovery successful. We opine that the developing countries can venture out to this kind of phosphorus recycling and substitute the high cost inorganic P-fertilizers with microalgal and cyanobacterial polyphosphates only if it is encouraged by the policy makers.

## Author contributions

CM identified the research problem, conceptualized and designed the work, performed the experiments, gathered data, interpreted, and analyzed the results and finally wrote the manuscript. RC isolated the strains for the work, performed the experiments and compiled data. KR as a supervisor, identified the research problem, conceptualized and designed the work, and wrote the manuscript.

### Conflict of interest statement

The authors declare that the research was conducted in the absence of any commercial or financial relationships that could be construed as a potential conflict of interest.
